# Kinetic Spectrophotometric Determination of Certain Cephalosporins in Pharmaceutical Formulations

**DOI:** 10.1155/2009/596379

**Published:** 2009-06-21

**Authors:** Mahmoud A. Omar, Osama H. Abdelmageed, Tamer Z. Attia

**Affiliations:** Analytical Chemistry Department, Faculty of Pharmacy, Minia University, 61519 Minia, Egypt

## Abstract

A simple, reliable, and sensitive kinetic spectrophotometric method was developed for determination of eight cephalosporin antibiotics, namely, Cefotaxime sodium, Cephapirin sodium, Cephradine dihydrate, Cephalexin monohydrate, Ceftazidime pentahydrate, Cefazoline sodium, Ceftriaxone sodium, and Cefuroxime sodium. The method depends on oxidation of each of studied drugs with alkaline potassium permanganate. The reaction is followed spectrophotometrically by measuring the rate of change of absorbance at 610 nm. The initial rate and fixed time (at 3 minutes) methods are utilized for construction of calibration graphs to determine the concentration of the studied drugs. The calibration graphs are linear in the concentration ranges 5–15 *μ*g mL^−1^ and 5–25 *μ*g mL^−1^ using the initial rate and fixed time methods, respectively. The results are validated statistically and checked through recovery studies. The method has been successfully applied for the determination of the studied cephalosporins in commercial dosage forms. Statistical comparisons of the results with the reference methods show the excellent agreement and indicate no significant difference in accuracy and precision.

## 1. Introduction

Cephalosporins consist of a fused *β*-lactam-Δ3-dihydrothiazine two-ring system, known as 7-aminocephalosporanic acids (7-ACAs) and vary in their side chain substituents at C3 (R2) and C7 (acylamido, R1). The chemical structure of the studied cephalosporins in this work is shown in Table 1. They are used for treatment of infection caused by both gram-negative and gram-positive bacteria [1, 2]. A wide variety of analytical methods have been reported for determination of cephalosporins in pure form, in pharmaceutical preparations, and in biological fluids. These methods include spectrophotometry [2–5], atomic absorption spectrophotometry [6], fluorometry [7–12], liquid chromatography [13–20], Micellar electrokinetic capillary chromatography [21, 22], chemiluminescence [23–28], potentiometric [29, 30], and polarographic [31–34] methods. Kinetic spectrophotometric methods became of great interest in chemical and pharmaceutical analyses [35]. The literature is still poor in analytical procedure based on kinetics, especially for determination of drug in commercial dosage forms. We aimed to improve on the current methods by employing the kinetic colorimetric oxidation of cephalosporins to increase selectivity, avoid interference of colored and/or turbidity background of samples and consequently determination of low concentration of the cited drugs as possible.

## 2. Experimental

### 2.1. Apparatus

Spectronic Genesys 2PC. Ultraviolet/Visible spectrophotometer (Milton Roy Co, USA) with matched 1 cm quartz cell was used for all measurements connected to IBM computer loaded with winspec application software.

### 2.2. Materials and Reagents

All the materials were of analytical reagent grade, and the solutions were prepared with double-distilled water. Samples of cephalosporin were generously supplied by their respective manufacturers and were used without further purification.

- Cephalexin monohydrate, Ceftazedime pentahydrate, and Cefuroxime sodium (Galaxowelcome Egypt, S.A.E, El Salam City, Cairo, Egypt).
- Cephapirin sodium, Cefazoline sodium (Bristol Myers- Squib Pharmaceutical Co., Cairo, Egypt).
- Cefotaxime sodium, cephradine, and Ceftriaxone sodium (EIPICO, Tenth of Ramadan City, Cairo, Egypt).

The purity of authentic samples was checked by UV assay methods and was not less than 99.26 ± 0.72.

- Potassium permanganate (Merck, Germany); 6 × 10^−3^ Molar solution was prepared by dissolving 100 mg in 100 mL of double-distilled water followed by boiling and filtration through sintered glass.

Potassium permanganate solution should be freshly prepared and its molarity was checked titrimetrically.

- Sodium hydroxide (El Nasr chemical co., Abuo Zabbel, Egypt); 0.75 M prepared by dissolving 3 g in 100 mL of double-distilled water.
- Methanol (Merck, Darmstadt, Germany).

### 2.3. Pharmaceutical Formulations

The following available commercial preparations were analyzed.

Ceporex tablets and vials, Fortam vials and Zinnat vials (Galoxowelcome Egypt, S.A.E, El Salam City, Cairo, Egypt), labeled to contain 250 mg cephalexin monohydrate per tablet and 500 mg cephalexin sodium equivalent to 500 mg cephalexin monohydrate per vial, 250 mg ceftazidime pentahydrate per vial, and 250 mg cefuroxime sodium per vial, respectively. Cefatrexyl vials, Totacef vials and Velosef tablets and suspensions (Bristol Myers-Squibb Pharmaceutical Co., Cairo, Egypt), labeled to contain 500 mg cephapirin sodium per vial, 500 mg cefazoline sodium per vial, and 500 mg cephradine per tablet and suspensions, respectively. Cefotax vials (EIPICO. Tenth of Ramadan City, Cairo, Egypt), labeled to contain 250 mg cefotaxime sodium per vial. Ceftriaxone vials (Novartis Pharma S.A.E, Cairo, Egypt), labeled to contain 500 mg ceftriaxone sodium per vial.

### 2.4. Preparation of Standard Solution

Stock solution containing 1 mg mL^−1^ of each cephalosporins was prepared in double-distilled water, working standard solutions containing 100 *μ*g mL^−1^ were prepared by suitable dilution of the stock solutions with double-distilled water.

### 2.5. Recommended Procedure for Cephalosporins Determination 

#### 2.5.1. Initial Rate Method

Aliquots of 50–150 *μ*g mL^−1^ of studied cephalosporins test solutions were pipetted into a series of 10 mL volumetric flask. 1.2 mL of sodium hydroxide solution (0.75 M) was added followed by 3.0 mL of potassium permanganate solution (6 × 10^−3^ M) to each flask and then diluted to the volume with double-distilled water at 30 ± 1°C. The content of mixture of each flask was mixed well and the increase in absorbance at 610 nm was recorded as a function of time for 15 minutes against reagent blank treated similarly. The initial rate of the reaction (*ν*) at different concentrations was obtained from the slope of the tangent to absorbance time curves. The calibration graphs were constructed by plotting the logarithm of the initial rate of the reaction (log   *ν*) versus logarithm of molar concentration of the studied drugs (log   *C*).

#### 2.5.2. Fixed Time Method

In this method, the absorbance of each sample solution at preselected fixed time (3 minutes) was accurately measured and plotted against the final concentration of the drug.

### 2.6. Determination of the Studied Drugs in Pharmaceutical Formulations 

#### 2.6.1. Procedure for Tablets

An accurately weighed amount equivalent to 100.0 mg of each drug from composite of 20 powdered tablets was transferred into a 100 mL volumetric flask. Dissolved in about 20 mL methanol, swirled, and sonicated for 10 minutes, the resultant mixture was filtered into round bottom flask for removal of any pharmaceutically “inert” ingredients (cellulose, disaccharides) that could be subject to oxidation by permanganate. The residue was washed thoroughly with about 5 mL methanol and the combined filtrate as well as washing solutions was subjected to evaporation under vacuum till dryness. The residue lifted was dissolved in about 20 mL distilled water and filtered into 100 mL volumetric flask. The filter paper was washed thoroughly with double distilled water, and then the combined filtrate as well as washing solutions was mixed well and completed to volume with the same solvent to obtain solution of 1.0 mg mL^−1^. The final solution was diluted quantitatively with the same solvent to obtain working standard solution of 100.0 *μ*g mL^−1^, then the general procedure was followed.

#### 2.6.2. Procedure for Capsules and Suspension

The contents of 20 capsules were evacuated and well mixed. Then an accurately weighed amount equivalent to 100.0 mg evacuated capsules or dry powder suspension of each drug was transferred into a 100 mL beaker, and then the procedure was continued as described under tablets.

#### 2.6.3. Procedure for Vials

A 100.0 mg quantity of each vial was transferred into a 100 mL volumetric flask, dissolved, and completed to the mark with double-distilled water to obtain solution of 1.0 mg mL^−1^. Further dilutions with double-distilled water were made to obtain sample solutions (100.0 *μ*g mL^−1^), then the general procedure was followed.

## 3. Results and Discussion

**Figure 1:**
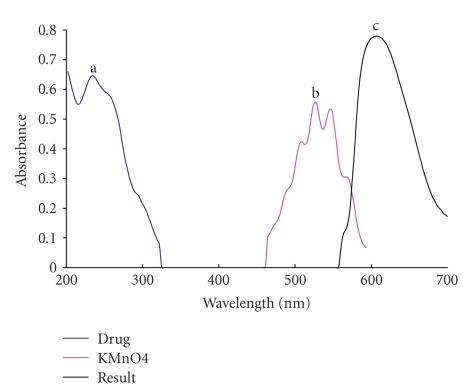
Absorption spectra of (a) alkaline potassium permanganate (6 × 10^−3^ M) (b) cefotaxime (15 *μ*g mL^−1^) and (c) the reaction product.

Potassium permanganate as strong oxidizing agent has been used in oxidimetric analytical method for determination of many compounds [36–39]. During the course of the reaction, the valence of manganese changes. The heptavalent manganese ion changes to the green color (Mn VI), while in neutral and acidic medium, the permanganate is further reduced to colorless (Mn II). The behavior of permanganate was the basis for its uses in development of spectrophotometric method. The absorption spectrum of aqueous potassium permanganate solution in alkaline medium exhibited an absorption band at 530 nm. The additions of any of the studied drugs to this solution produce a new characteristic band at 610 nm (Figure 1). This band is due to formation of manganate ion, which resulted from the oxidation of cephalosporin by potassium permanganate in alkaline medium. The intensity of the color increases with time; therefore a kinetically based method was developed for determination of cephalosporins in their pharmaceutical dosage formulations. The different variables that affect the formation of manganate ion were studied and optimized.

### 3.1. Effect of Potassium Permanganate Concentration

**Figure 2:**
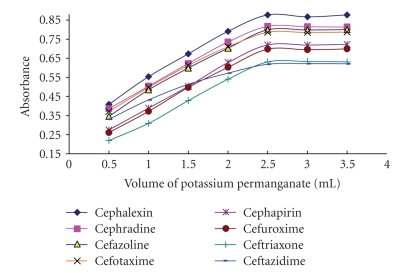
Effect of potassium permanganate (3 × 10^−6^) on the reaction between the investigated cephalosporins (15 *μ*g mL^−1^) and alkaline potassium permanganate.

The absorbance increases substantially with increasing the concentration of potassium permanganate (Figure 2). Maximum absorbance was obtained when 2.5 mL of 6 × 10^−3^ M of potassium permanganate was used. Thus, the adoption of 3 mL of potassium permanganate in the final solution proved to be adequate for the maximum concentration of cephalosporin used in determination process (the concentration of the final assay was 1.8 × 10^−3^ M).

### 3.2. Effect of Sodium Hydroxide Concentration

**Figure 3:**
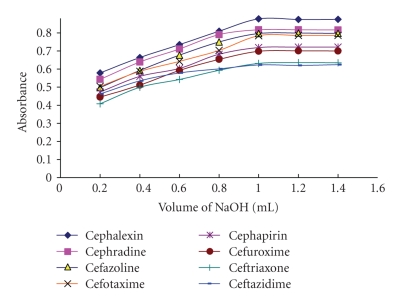
Effect of sodium hydroxide concentration (0.75 M) on the reaction between the investigated cephalosporins (15 *μ*g mL^−1^) and alkaline potassium permanganate.

Maximum absorption was obtained when 1 mL of 0.75 M NaOH was used. Over this volume no change in absorbance could be detected. So 1.2 mL of 0.75 M of NaOH was used as an optimum value (Figure 3).

### 3.3. Effect of Temperature

At room temperature the reaction rate increases substantially with time, although heating the solution was found to increase the rate of the reaction however but MnO_2_ was precipitated, therefore room temperature was selected as the optimum temperature.

### 3.4. Stoichiometry and Reaction Mechanism

**Scheme 1:**
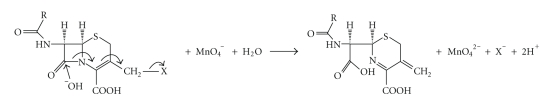


**Figure 4:**
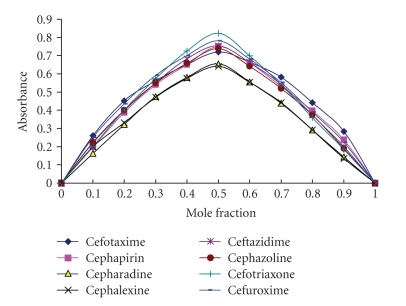
Job's plots of continuous variation between potassium permanganate and the studied drugs.

The stoichiometric ratio between potassium permanganate and each of investigated cephalosporins was determined by Job's method [40, 41] and was found to be 1 : 1 (Figure 4). Cephalosporins were found to be susceptible for oxidation with alkaline potassium permanganate producing a green color peaking at 610 nm. Therefore, the reaction mechanism is proposed on the basis of the literature background (39) and our experimental study as shown in Scheme 1.

The opening of the *β*-lactam ring by the hydroxyl ion proceeds via intermediate and result in the formation of cephalosporoic acid and the intermediate formation is the rate limiting step.

### 3.5. Kinetic of the Reaction

**Figure 5:**
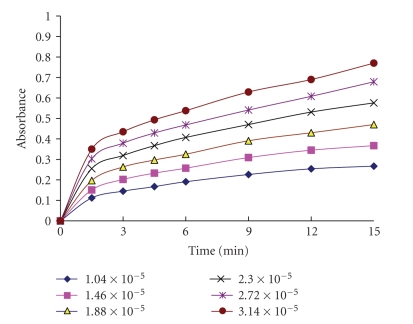
Absorption versus time for the reaction between cefotaxime (different molar concentrations) and KMnO_4_ (1.8 × 10^−3^ mol L^−1^).

**Figure 6:**
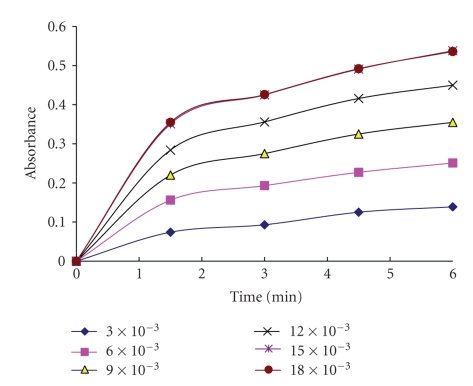
Absorption versus time for the reaction between cefotaxime 3.14 × 10^−5^ mol L^−1^ and KMnO_4_ (different molar concentrations).

Under the optimum conditions, the absorbance time curves of investigated cephalosporins with potassium permanganate reagent were constructed (Figures 5, 6 for cefotaxime as a representative example). The initial rate of the reaction was determined from the slope of tangents of the absorption time curves. The order of the reaction with respect to permanganate was determined by studying the reaction at different concentrations of permanganate with fixed concentration of investigated cephalosporins. The plot of initial rate (Δ*A*/Δ*t*) against initial absorbance was linear passing through origin indicating that the initial order of the reaction with respect to permanganate was 1. The order with respect to investigated cephalosporins was evaluated by measuring the rate of the reaction at several concentrations of cephalosporins at a fixed concentration of permanganate reagent. This was done by plotting the logarithm of initial rate of the reaction versus logarithm of molar concentration of investigated cephalosporins and was found to be 1. However under the optimized experimental conditions, the concentrations of cephalosporins were determined using relative excess amount of potassium permanganate and sodium hydroxide solutions. Therefore pseudo-zero-order conditions were obtained with respect to their concentrations.

### 3.6. Quantitation Methods 

#### 3.6.1. Initial Rate Method

The initial rate of the reaction would follow pseudo-first-order and were found to obey the following equation:

(1) $$\nu =\frac{\Delta A}{\Delta t}=K^{\prime }C^{n},$$

where *ν* is the reaction rate, *A* is the absorbance, *t* is the measuring time, *K*′ is the pseudo-first-order rate constant, *C* is the molar concentration of cephalosporins, and *n* is the order of the reaction. The logarithmic form of the above equation is written as follows:

(2) $$\text{Log}\;\nu =\log\;\;\frac{\Delta A}{\Delta t}=\log\;\;\;K^{\prime }+n\;\log\;\;\;C.$$

Regression analysis using the method of least square was performed to evaluate the slopes, intercepts, and correlation coefficient.

The analytical parameters and results of regression analysis are given in Table 2. The value of *n*(≈1) in the regression equation confirmed that the reaction of cephalosporins with the potassium permanganate was pseudo-first-order with respect to cephalosporins concentration. The limits of detection (LOD) were calculated and results obtained confirmed good sensitivity of the proposed method and consequently their capabilities to determine low amount of cephalosporins.

#### 3.6.2. Fixed Time Method

In this method, the absorbance of the reaction solution containing varying amount of cephalosporins was measured at preselected fixed time. Calibration plots of absorbance versus the concentration of cephalosporins at fixed time were established for each investigated cephalosporins. The regression equation, correlation coefficients, and detection limits are given in Table 3. The lowest detection limit was obtained at fixed time of 15 minutes. However the fixed time of 3 minutes showed a wider concentration range for quantification. 

According to international conference of harmonization (ICH) guideline for validation of analytical procedures [42], the detection limit is not required to be part of validation procedure for assay. Therefore on the basis of wider concentration range and less time of analysis, the fixed time of 3 minutes was recommended for determination.

### 3.7. Validation of the Proposed Method

**Figure 7:**
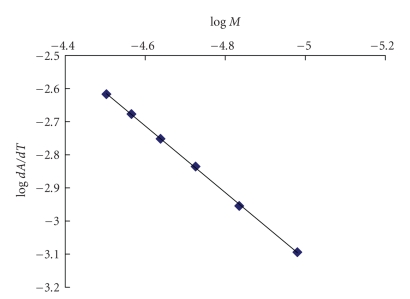
Calibration Plot of logarithm rate of the reaction against logarithm molar concentration of cefotaxime for initial rate method.

**Figure 8:**
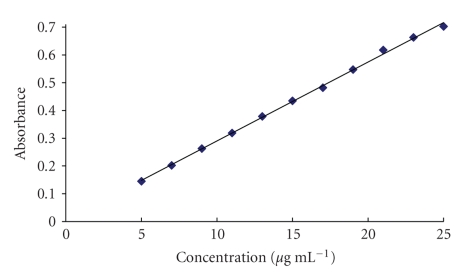
Calibration plot of absorbance versus the concentration of cefotaxime at preselected fixed time of 3 minutes.

Concentration range [42] is established by confirming that the analytical procedure provides a suitable degree of precision, accuracy, and linearity when applied to the sample containing amount of analyte within or at the extreme of the specified range of the analytical procedure [43, 44]. In this work, concentrations ranging from 7.8 × 10^−6^ M to 4.31 × 10^−5^ M were studied for the investigated drugs in the initial rate method and concentration ranging from 5 to 25 *μ*g mL^−1^ were studied for the investigated drugs in the fixed time method (at preselected fixed time of 3 minutes). The whole set of experiments were carried out through this range to ensure the validation of the proposed procedure. Linear calibration graphs were obtained for all the studied drugs by plotting the logarithm of initial rate of the reaction versus logarithm of molar concentration of analyte in the sample (in initial rate method) within the specified range (Figure 7):

(3) $$\text{Log}\;\nu =\log\;\;\;\frac{\Delta A}{\Delta t}\;=\log\;\;\;K^{\prime }+n\;\log\;\;\;C\;\left(\text{where}\;n\approx 1\right)$$

or by plotting the absorbance of the studied drugs versus the drug concentration (in fixed time method) within the specified range (Figure 8). 

Linearity was studied for both initial rate and fixed time method indicated by the values of correlation coefficient (*r*) and determination coefficient (*r* ^2^) for both method (Tables 2 and 3).

*Accuracy* [44] was checked at three concentration levels within the specified range, six replicate measurements were recorded at each concentration levels. The results were recorded as percentage recovery ± standard deviation (Tables 4 and 5).

*Precision* [44] was checked at three concentration levels, eight replicate measurements were recorded at each concentration level. The results are summarized in (Table 6). The calculated relative standard deviation were all below 2.2% indicating excellent precision of the proposed procedure at both level of repeatability and intermediate precision.

*Specificity and interference* the proposed procedure was performed in visible region away from the UV-absorption region of investigated drugs (228–300 nm), and the interference by reducing sugars in tablets was eliminated by extraction with methanol prior to analysis. However interference was observed from L-arginine formulated with cephradine in velosef vial. Therefore, the proposed procedure could not be used for analysis of cephradine in presence of L-arginine, except after separation by a suitable separation technique. 

*Limit of detection (LOD*) [42] was calculated based on standard deviation of response and the slope of calibration curve [43]. The limit of detection was expressed as [44]

(4) $$\text{LOD}=\frac{3\sigma }{S},\;$$

where *σ* is the standard deviation of intercept. *S* is the slope of calibration curve.

The results were summarized in (Tables 2 and 3) indicating good sensitivity of the proposed method. According to USP *XXV* validation guidelines [44], the calculated LOD values should be further validated by laboratory experiments. In our work, good results were obtained where the calculated drug concentration by LOD equations were actually detected in these experiments.

*Limit of quantitation (LOQ) was* calculated based on standard deviation of intercept and slope of calibration curve. In this method, the limit o quantitation is expressed as [44]

(5) $$\text{LOQ}=\frac{10\sigma }{S}.$$

The results were summarized in (Tables 2 and 3) indicating good sensitivity of the proposed method. According to USP XXV validation guidelines [45], the calculated LOQ values should be further validated by laboratory experiments. In our work, good results were obtained where the calculated drug concentration by LOQ equations were actually quantitated in these experiments.

### 3.8. Application to Pharmaceutical Dosage Forms

The initial rate and fixed time methods of the proposed kinetic spectrophotometric method for determination of investigated cephalosporins have been tested on commercial pharmaceutical dosage forms. The concentration of investigated cephalosporins was computed from its responding regression equations. The results of proposed method (initial rate and fixed time) were statistically compared with those of reported methods [3–5], in respect to accuracy and precision. The obtained mean recovery values were 99.2–100.67 ± 0.6226–1.69% (Table 7), which ensures that there is no interference of other additives present in the studied formulations.

In the *t*- and *F*-tests, no significant differences were found between the calculated and theoretical values of both the proposed and the reported methods at 95% confidence level. This indicates good precision and accuracy in the analysis of investigated cephalosporins in pharmaceutical dosage forms.

## 4. Conclusion

The initial rate and fixed time methods can be easily applied for determination of investigated cephalosporins in pure and dosage forms that do not require elaborate treatment and tedious extraction of chromophore produced. The proposed method (initial rate or fixed time) is sensitive enough to enable determination of lower amounts of drug, these advantages encourage the application of proposed method in routine quality control of investigated cephalosporins in industrial laboratories. Finally our method provides advantages of improving selectivity, avoiding interference of colored and/or turbidity background of samples because it measures the increase in absorbencies with time against blank treated similarly. 

## Figures and Tables

**Table 1:** Structural formula of the studied cephalosporins.

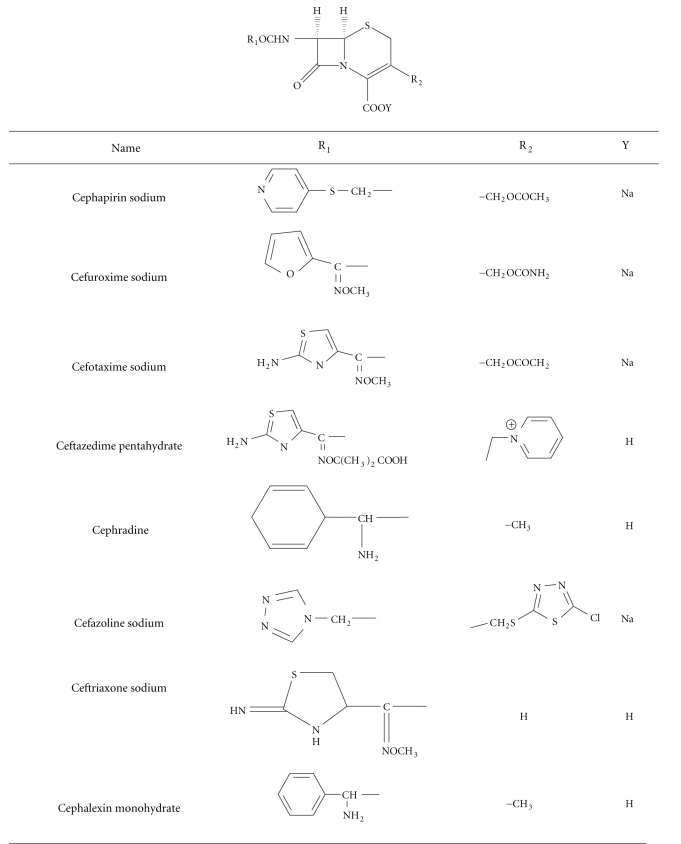

**Table 2:** Analytical parameters for the initial rate method for determination of investigated cephalosporins with alkaline potassium permanganate.

Investigated cephalosporin	Linear range,	Least square equation		Correlation	
	M × 10^−5^	log *V* = log *K*′ + *n* log *C*		coefficient	LOD *μ*g mL^−1^
	(*μ*g mL^−1^)	Intercept (log *K*′)	Slope (*n*)	(*r*)	
Cefotaxime	1.04 to 3.14	1.918	1.007	0.9999	0.121
	(5–15)				
Cephapirin	1.18 to 3.54	1.866	1.010	0.9999	0.117
	(5–15)				
Cephradine	1.43 to 4.29	1.788	0.9942	0.9997	0.162
	(5–15)				
Cephalexin	1.43 to 4.31	1.856	1.001	0.9996	0.19
	(5–15)				
Ceftazidime	0.78 to 2.33	2.044	1.046	0.9995	0.233
	(5–15)				
Cefazoline	1.1 to 3.3	1.832	0.9918	0.9992	0.28
	(5–15)				
Ceftriaxone	0.9 to 2.7	1.884	1.009	0.9994	0.241
	(5–15)				
Cefuroxime	1.17 to 3.53	1.985	1.045	0.9996	0.209
	(5–15)				

**Table 3:** Analytical parameters for fixed time method of the kinetic spectrophotometric parameter for determination of investigated cephalosporins.

Reaction time	Linear range (*μ*g mL^−1^)	Intercept (*a*)	Standard deviation of intercept(*S*_*a*_)	Slope (*b*)	Standard deviation of slope (*S*_*b*_)	Correlation coefficient (*r*)	LOD (*μ*g mL^−1^)
Cefotaxime							
3	5–25	0.00568	0.005827	0.02845	0.0003607	0.9993	0.6144
6	5–19	0.01698	0.005317	0.03472	0.0004140	0.9996	0.4590
9	5–17	0.02618	0.005363	0.04004	0.0004582	0.9997	0.4018
12	5–17	0.02880	0.005112	0.04516	0.0004368	0.9998	0.3395
15	5–17	0.01995	0.005112	0.05102	0.0004368	0.9998	0.3005

Cephapirin							
3	5–25	0.00105	0.005027	0.02801	0.0003088	0.9995	0.5384
6	5–19	0.03286	0.004621	0.03176	0.0003598	0.9996	0.4364
9	5–17	0.05639	0.003562	0.03525	0.0003043	0.9998	0.30314
12	5–17	0.08327	0.003653	0.03791	0.0003121	0.9998	0.289
15	5–17	0.09054	0.003790	0.04196	0.0003238	0.9999	0.27097

Cephradine							
3	5–25	0.01464	0.009607	0.03216	0.0005901	0.9985	0.896
6	5–19	0.02004	0.007665	.03856	0.0005967	0.9993	0.596
9	5–17	0.04891	0.005234	0.04184	0.0004471	0.9997	0.375
12	5–17	0.06645	0.004959	0.04566	0.0004237	0.9998	0.3258
15	5–17	0.07721	0.005174	0.04907	0.0004420	0.9998	0.3163

Cephalexin							
3	5–25	0.01573	0.006484	0.03518	0.0003983	0.9994	0.5529
6	5–19	−0.0067	0.007189	0.04353	0.0005580	0.9995	0.495
9	5–17	−0.0012	0.005694	0.04841	0.0004865	0.9997	0.3528
12	5–17	−0.0040	0.005204	0.05354	0.0004446	0.9998	0.2915
15	5–17	−0.0015	0.005412	0.05821	0.0004624	0.9998	0.2789

Ceftazidime							
3	5–25	−0.0029	0.003859	0.01905	0.0002371	0.9993	0.6077
6	5–19	0.03039	0.004045	0.02324	0.0003149	0.9994	0.52216
9	5–17	0.04486	0.003966	0.02871	0.0003388	0.9997	0.41442
12	5–17	0.06577	0.003875	0.03270	0.0003311	0.9997	0.3555
15	5–17	0.07993	0.003785	0.03636	0.0003234	0.9998	0.3128

Cefazoline							
3	5–25	0.00027	0.006894	0.02935	0.0004235	0.9991	0.70466
6	5–19	0.02604	0.006741	0.03439	0.0005246	0.9993	0.588
9	5–17	0.03064	0.006040	0.04064	0.0005161	0.9996	0.4458
12	5–17	0.04073	0.005576	0.04566	0.0004764	0.9997	0.366
15	5–17	0.03973	0.005958	0.05038	0.0005090	0.9997	0.3547

Ceftriaxone							
3	5–25	−0.0024	0.004203	0.02274	0.0002582	0.9994	0.55448
6	5–19	0.02739	0.004303	0.02770	0.0003329	0.9996	0.466
9	5–17	0.01350	0.004163	0.03307	0.0003557	0.9997	0.377
12	5–17	0.02534	0.004423	0.03741	0.0003779	0.9997	0.3546
15	5–17	0.03982	0.003818	0.03939	0.0003262	0.9998	0.2907

Cefuroxime							
3	5–25	−0.0065	0.005980	0.02629	0.0003674	0.9991	0.68238
6	5–19	0.02696	0.005472	0.02994	0.00004260	0.9994	0.54829
9	5–17	0.04286	0.005334	0.03471	0.0004557	0.9996	0.4610
12	5–17	0.05480	0.004229	0.03845	0.0003613	0.9998	0.32996
15	5–17	0.06130	0.005614	0.04266	0.0004796	0.9997	0.2747

**Table 4:** Evaluation of accuracy of the analytical procedure using initial rate method.

Drug	Recovery %*		
	5.0 *μ*g mL^−1^	9.0 *μ*g mL^−1^	15.0 *μ*g mL^−1^
Cefotaxime	99.8 ± 1.061	100.06 ± 0.7136	99.89 ± 0.6435
Cephapirin	100.29 ± 1.071	99.68 ± 0.7181	100.22 ± 0.7661
Cephradine	100.74 ± 0.8237	99.98 ± 0.6211	99.87 ± 0.6870
Cephalexin	99.41 ± 0.7567	99.78 ± 0.5612	99.73 ± 0.4031
Ceftazidime	100.14 ± 0.9817	100.16 ± 0.8680	99.18 ± 0.8714
Cefazoline	99.89 ± 1.243	100.87 ± 0.8745	100.4 ± 0.6999
Ceftriaxone	99.31 ± 1.242	99.99 ± 0.9167	100.67 ± 0.7308
Cefuroxime	100.21 ± 1.168	100.18 ± 0.8334	100.69 ± 0.9595

*Mean of 6 replicate ± SD.

**Table 5:** Evaluation of accuracy of the analytical procedure using fixed time method.

Drug	Recovery %*		
	5.0 *μ*g mL^−1^	9.0 *μ*g mL^−1^	15.0 *μ*g mL^−1^
Cefotaxime	98.75 ± 0.8183	100.98 ± 0.8118	100.75 ± 0.6424
Cephapirin	100.28 ± 1.335	99.35 ± 0.7414	100.36 ± .7051
Cephradine	100.76 ± 1.164	101.87 ± 0.9712	100.58 ± 0.8675
Cephalexin	98.59 ± 0.839	100.09 ± 0.5185	100.77 ± 0.6852
Ceftazidime	99.66 ± 1.485	100.15 ± 0.8789	99.88 ± 0.6548
Cefazoline	99.63 ± 1.262	101.42 ± 0.5617	99.44 ± 0.6155
Ceftriaxone	99.47 ± 0.9185	100.59 ± 0.9242	99.94 ± 0.5474
Cefuroxime	99.57 ± 0.9475	99.05 ± 0.9773	100.85 ± 0.7630

*Mean of 6 replicate ± SD.

**Table 6:** Evaluation of precision of the initial rate and fixed time methods of the proposed kinetic spectrophotometric method for determination of investigated cephalosporins.

Drug	Amount taken	Recovery (% ± SD)	
	(*μ*g mL^−1^)	Initial rate method	Fixed time method
Cefotaxime	5	100.1 ± 1.072	99.82 ± 0.7246
	9	99.88 ± 0.6859	100.9 ± 0.7086
	15	99.95 ± 0.5668	100.6 ± 0.6286
Cephapirin	5	100.16 ± 1.015	100.29 ± 1.144
	9	99.68 ± 0.6840	99.2 ± 0.7160
	15	100.31 ± 0.7624	100.44 ± 0.6225
Cephradine	5	100.61 ± 0.8049	100.53 ± 1.125
	9	99.94 ± 0.5594	101.84 ± 0.9913
	15	99.70 ± 0.6116	100.51 ± 0.9054
Cephalexin	5	99.56 ± 0.7435	98.64 ± 0.7306
	9	99.78 ± 0.5811	100.04 ± 0.4796
	15	99.76 ± 0.4575	100.78 ± 0.6091
Ceftazidime	5	100.2 ± 1.002	99.53 ± 1.309
	9	100.19 ± 0.7958	100.6 ± 0.9820
	15	99.43 ± 0.8901	99.88 ± 0.6205
Cefazoline	5	99.88 ± 1.066	99.64 ± 1.082
	9	101.07 ± 0.8312	101.45 ± 0.4871
	15	100.44 ± 0.7346	99.46 ± 0.5538
Ceftriaxone	5	99.28 ± 1.154	99.34 ± 0.9175
	9	99.99 ± 0.8687	100.54 ± 0.8347
	15	100.76 ± 0.6403	99.98 ± 0.4933
Cefuroxime	5	100.19 ± 1.19	99.52 ± 0.8834
	9	100.4 ± 0.8276	99.23 ± 0.9490
	15	100.39 ± 0.6692	100.94 ± 0.408

**Table 7:** Determination of studied drugs in their pharmaceutical dosage forms using initial rate and fixed time methods.

Drug	Pharmaceutical dosage form	Proposed methods ± SD (*n* = 5)		Reported methods ± SD
		Initial rate	Fixed time	(*n* = 5)
Cefotaxime	Cefotax vials	100.67 ± 0.9359	99.98 ± 1.146	100.58 ± 0.9753
		*t* = 0.1423*	*t* = 0.8914	
		*F* = 1.086*	*F* = 1.382	
Cephapirin	Cefatrexyl vials	100.57 ± 0.9274	99.87 ± 1.112	100.17 ± 1.208
		*t* = 0.5901	*t* = 0.4193	
		*F* = 1.698	*F* = 1.180	
Cephradine	Velosef suspension	99.85 ± 1.274	99.4 ± 1.699	99.89 ± 1.190
		*t* = 0.1227	*t* = 0.5303	
		*F* = 1.346	*F* = 2.039	
Cephradine	Velosef capsuls	100.1 ± 1.071	100.56±0.6226	100.25 ± 0.7994
		*t* = 0.2477	*t* = 0.6753	
		*F* = 1.793	*F* = 1.649	
Cephalexin	Ceporex vials	99.69 ± 1.0	100.43 ± 0.918	100.19 ± 0.9310
		*t* = 0.8248	*t* = 0.4069	
		*F* = 1.154	*F* = 1.027	
Cephalexin	Ceporex tablets	99.93 ± 1.370	100.06 ± 1.647	99.98 ±1.112
		*t* = 0.06844	*t* = 0.08551	
		*F* = 1.518	*F* = 2.194	
Ceftazidime	Fortum vials	99.2 ± 1.261	99.74 ± 1.212	99.78 ± 0.6750
		*t* = 0.9192	*t* = 0.7738	
		*F* = 3.490	*F* = 3.222	
Cefazoline	Totacef vials	99.69 ± 1.586	99.35 ± 1.059	99.25 ± 1.379
		*t* = 0.4724	*t* = 0.1234	
		*F* = 1.321	*F* = 1.695	
Ceftriaxone	Ceftriaxone vials	99.92 ± 1.292	99.43 ± 1.185	100.04 ±0.9742
		*t* = 0.1658	*t* = 0.8951	
		*F* = 1.760	*F* = 1.479	
Cefuroxime	Zinnat vials	100.31 ± 1.091	99.82 ± 1.105	100.33 ± 0.7431
		*t* = 0.0373	*t* = 0.8533	
		*F* = 2.157	*F* = 2.209	

*Tabulated value at 95% confidence limit; *t* = 2.306 and *F* = 6.388.
